# Characterization of three *TRAPPC11* variants suggests a critical role for the extreme carboxy terminus of the protein

**DOI:** 10.1038/s41598-019-50415-6

**Published:** 2019-10-01

**Authors:** Miroslav P. Milev, Daniela Stanga, Anne Schänzer, Andrés Nascimento, Djenann Saint-Dic, Carlos Ortez, Daniel Natera-de Benito, Desiré González Barrios, Jaume Colomer, Carmen Badosa, Cristina Jou, Pia Gallano, Lidia Gonzalez-Quereda, Ana Töpf, Katherine Johnson, Volker Straub, Andreas Hahn, Michael Sacher, Cecilia Jimenez-Mallebrera

**Affiliations:** 1 Concordia University, Department of Biology, Montreal, Quebec Canada; 2 Institute of Neuropathology, Justus Liebig University Giessen, Giessen, Germany; 3 Neuromuscular Unit, Neuropaediatrics Department, Hospital Sant Joan de Déu, Institut de Recerca Sant Joan de Déu, Barcelona, Spain; 4 U705 and U703 Center for Biomedical Research on Rare Diseases (CIBERER), Instituto de Salud Carlos III, Madrid, Spain; 5 Servicio de Pediatría, Hospital Universitario Nuestra Señora de Candelaria, Santa Cruz de Tenerife, Spain; 6 Pathology Department and Biobank, Hospital Sant Joan de Déu, Institut de Recerca Sant Joan de Déu, Barcelona, Spain; 7 Servicio de Genética, Hospital de la Santa Creu i Sant Pau, Barcelona, Spain; 8 The John Walton Muscular Dystrophy Research Centre, Institute of Genetic Medicine, Newcastle University and Newcastle Hospitals NHS Foundation Trust, Newcastle-upon-Tyne, UK; 9 Institute of Cellular Medicine, Newcastle University, Newcastle-upon-Tyne, UK; 10 Department of Child Neurology, Justus Liebig University Giessen, Giessen, Germany; 11 McGill University, Department of Anatomy and Cell Biology, Montreal, Quebec Canada

**Keywords:** Medical genomics, Neurodevelopmental disorders, Genetics research

## Abstract

TRAPPC11 was identified as a component of the TRAPP III complex that functions in membrane trafficking and autophagy. Variants in *TRAPPC11* have been reported to be associated with a broad spectrum of phenotypes but all affected individuals display muscular pathology. Identifying additional variants will further our understanding of the clinical spectrum of phenotypes and will reveal regions of the protein critical for its functions. Here we report three individuals from unrelated families that have bi-allellic *TRAPPC11* variants. Subject 1 harbors a compound heterozygous variant (c.1287 + 5G > A and c.3379_3380insT). The former variant results in a partial deletion of the foie gras domain (p.Ala372_Ser429del), while the latter variant results in a frame-shift and extension at the carboxy terminus (p.Asp1127Valfs*47). Subjects 2 and 3 both harbour a homozygous missense variant (c.2938G > A; p.Gly980Arg). Fibroblasts from all three subjects displayed membrane trafficking defects manifested as delayed endoplasmic reticulum (ER)-to-Golgi transport and/or a delay in protein exit from the Golgi. All three individuals also show a defect in glycosylation of an ER-resident glycoprotein. However, only the compound heterozygous subject displayed an autophagic flux defect. Collectively, our characterization of these individuals with bi-allelic *TRAPPC11* variants highlights the functional importance of the carboxy-terminal portion of the protein.

## Introduction

The mammalian transport protein particle (TRAPP) II and III are a family of related multisubunit complexes that are composed of a common core of subunits and contain complex-specific proteins that impart their differing functions. The TRAPPC11 protein was identified as a component that associates with TRAPP III^1,2^. This complex was originally reported to play an as yet undefined role in membrane trafficking in the biosynthetic pathway. More recently, a role for the TRAPP III-specific subunits in autophagy was proposed^3–6^. Specifically, the TRAPPC11 protein was shown to function upstream of autophagosome sealing, while TRAPPC12, another component of this complex, functions prior to autophagosome-lysosome fusion^6^. Although the yeast TRAPP III complex has been implicated as a guanine nucleotide exchange factor (GEF) for Ypt1^7–9^, the Rab target of the human TRAPP III complex is still unclear. Interestingly, *Drosophila* TRAPP III was shown to be capable of nucleotide exchange on Rab1^10^. Whether all of the TRAPP III proteins function independently in other cellular processes is still unclear but two subunits, TRAPPC11 and TRAPPC12, appear to function in *N*-linked glycosylation (TRAPPC11), and mitosis and recruitment of the COP II coat (TRAPPC12)^11–13^. Thus, elucidating the roles of TRAPP III proteins both within and outside of the complex is important to better understand their cellular functions. In the case of TRAPPC11 and other TRAPP proteins, it is possible that regions or domains of the protein may play a role in different cellular pathways.

To date, a number of variants in the genes encoding TRAPP proteins have been described^14^. While variants in a core subunit called TRAPPC2 exclusively result in a skeletal defect, those in TRAPPC11 result in neuromuscular defects as well as a broad spectrum of other clinical features including scoliosis, alacrima, steatosis, intellectual deficit and cerebral atrophy^9,15–20^. This may be due to the various processes that are impacted by TRAPPC11 within the cell.

The domain structure of TRAPPC11 indicates several regions that may be involved in either protein-protein interactions or may be important for its function. For example, a region spanning amino acids 263–561, referred to as the foie gras domain, is well-conserved through evolution, suggesting a critical function for this domain. Variants within this domain have been reported including an in-frame deletion^9,15,18,20^, further highlighting the importance of this region. In addition, a well-conserved 60-amino acid stretch near the carboxy terminus referred to as the gryzun domain has also been noted, although its functional significance remains unknown. Most studies on TRAPPC11 variants have assessed membrane trafficking defects and Golgi morphology and found either one or both to be defective (reviewed in^14^).

The mapping of natural variants in a protein is useful in elucidating its function and allowing clinicians and researchers to better predict the cellular consequences of new variants. In the case of TRAPP proteins, a missense variant in TRAPPC2 that changes aspartate 47 to tyrosine was shown to affect the interaction between TRAPPC2 and both TRAPPC8 and TRAPPC9^21,22^. A similar missense variant in TRAPPC2L results in elevated levels of the active form of the GTPase Rab11^23^. In other instances, such as for described variants in TRAPPC9^14^, it remains unclear how they affect the function of the protein. Thus, identifying new variants and individuals with previously-described variants will further our understanding of the importance of specific regions of TRAPP proteins.

Here, we describe three individuals with either homozygous or compound heterozygous variants in *TRAPPC11*. All three individuals have variants at or near the carboxy-terminus of the protein. The compound heterozygous individual also has an in-frame deletion in a highly-conserved central region of the protein that was previously reported^15^. The purpose of this study was to analyze in detail the effects of these genetic defects on protein function. The clinical and cell biological features for each individual support the notion that the carboxy-terminus of the protein is critical for biological function.

## Results

### Clinical summary and molecular genetics

A summary of the clinical features for all the subjects in this study can be found in Table 1.

**Table 1:** Clinical features of subjects in this study with *TRAPPC11* variants.

	S1	S2	S3	S4
Variant (s)	c.1287 + 5G > A/c.3379_3380insT	c.2938G > A/p.Gly980Arg	c.2938G > A/p.Gly980Arg	c.1287 + 5G > A
Age of onset	first year of life	first year of life	3–4 years	not reported, family history of early onset
Muscle symptoms	axial hypotonia	axial hypotonia	axial hypotonia	none
Motor ability	unable to sit or stand	unable to sit or stand	walks with support	walks without support
muscle biopsy	minimal myopathic changes	myopathic	myopathic	not performed
CK (IU/l)	350–2817	600–1800	400–1500	700–1000
Hyperkinesia	yes	no	no	no
Spasticity	yes	no	no	no
Choreiform movements	yes	no	no	generalized
Other neurological symptoms	no	no	no	ataxia, primary generalized seizures
Intellectual disability	yes	yes	yes	moderate
microcephaly	yes	no	no	no
Neuroimaging	cerebral atrophy and reduced white matter volume	not done	mild cortical atrophy	mild cerebral atrophy
Ocular involvement	no	no	cataract	no
Hepatic involvement	no	no	reduced cholinesterase levels	no

**Figure 1:**
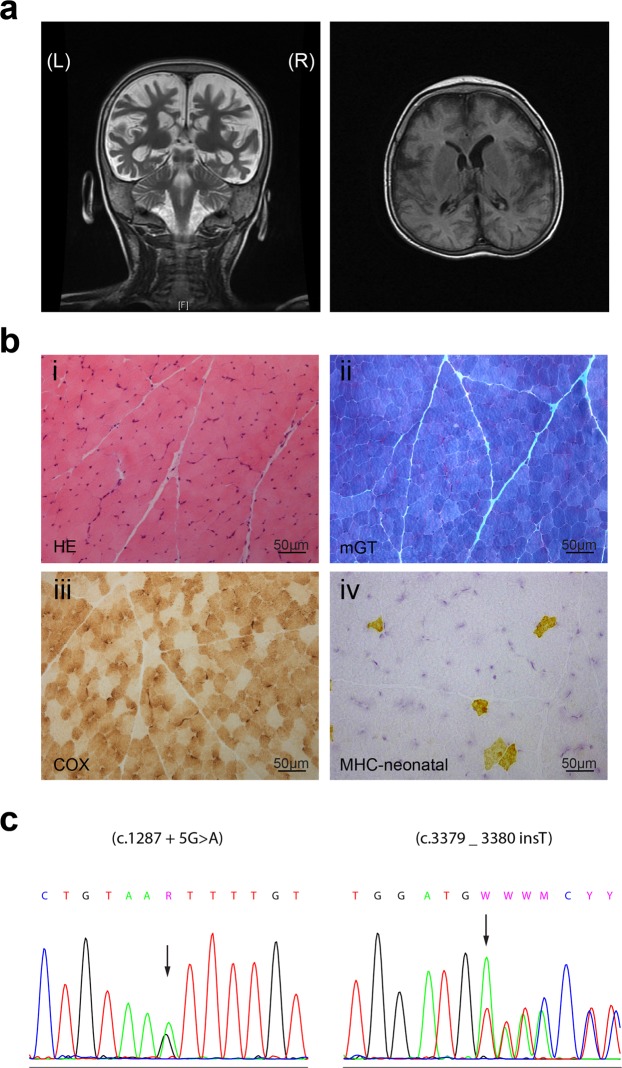
Brain and muscle abnormalities in subject S1 at age 3 and 4 years. (**a**) Brain magnetic resonance imaging of the individual at the age of 3. Note cortical atrophy and reduced white matter volume in both coronal T2-weighted (left panel) and axial T1-weighted (right panel) images. (**b**) Muscle biopsy was performed at the age of 4 years and showed minimal myopathic changes consisting of mild variation in muscle fibre diameter (i, ii), a population of fibres with weak cytochrome c activity (iii) and a few fibres of normal size positive for neonatal myosin (iv).

Subject S1 is a male infant born at 37 weeks of gestation. He was the first child of a healthy, young Caucasian couple. The family history was unremarkable with no similarly affected individuals, consanguinity, birth defects, or miscarriages. The pregnancy history was normal. The infant’s birth weight was 2948 g, length was 48 cm, and head circumference was 31.5 cm, all within the standard range (10^th^–90^th^ centiles) for male Spanish neonates. Developmental delay was noted since head control was not acquired until 4 months old. The subject presented with a febrile status epilepticus at five months old. Hypotonia, spasticity, and abnormal involuntary movements, including hyperkinesia and choreiform movements, appeared during the first year of life. Ability to sit, stand, or say words was never acquired. Cerebral atrophy and reduced white matter volume were observed from the first magnetic resonance imaging study (Fig. 1a). Serum creatine kinase (CK) levels were persistently elevated, ranging from 350 to 2817 IU/l (normal value < 190 IU/l). Muscle biopsy was performed at the age of 4 years and showed minimal myopathic changes consisting of mild variation in muscle fibre diameter, a population of fibres with weak cytochrome c activity and a few fibres of normal size positive for neonatal myosin indicating fiber regeneration (Fig. 1b). Intracellular lipids and glycogen were within normal limits. Immunohistochemistry for sarcolemmal proteins was normal. Alpha-dystroglycan glycosylation by means of immunofluorescence (clone IIH6 and VIA4-1) and immunoblot (IIH6) was not significantly reduced compared to the normal control analysed in parallel (not shown).

On recent assessment the subject was 9 years old. His height was 121 cm (centile < 1, −2.45 SD), weight was 21.3 kg (centile 6, −1.58 SD) and head circumference was 44.5 cm (centile < 1, −6.5 SD). Poor eye contact, axial hypotonia, spasticity, and choreiform movements persisted. He was unable to sit independently. A remarkable cerebral atrophy remained in successive magnetic resonance studies. There was no evidence of any clinical seizure since the febrile status. Successive EEGs did not register electrical seizures or epileptiform activity. Antiepileptic treatment with valproic acid was initiated after the first febrile status at 5 months and stopped at the age of 4 years.

Because of global hypotonia and elevated CK, the subject was included in the MYO-SEQ project (undiagnosed myopathies/limb girdle muscular dystrophies presented with marked muscle with elevated CK). Whole exome sequencing revealed that S1 harbours two heterozygous variants in *TRAPPC11*. The first is an extended splice site variant, c.1287 + 5G > A (GRCh37/hg19 chr4:184605212), that is rare in both the Genome Aggregation Database (gnomAD) and in the Exome Aggregation Consortium (ExAC) database control population (0.005% and 0.002%, respectively, with no homozygotes) and has a CADD PHRED score^24^ of 15.64 (Table 2). The variant, which results in a 58 amino acid in-frame deletion (p.Ala372_Ser429del), has been previously reported in homozygosity to be associated with myopathy, infantile hyperkinetic movements, ataxia and intellectual disability in five individuals of Hutterite descent^15^. The second variant results in a frameshift, c.3379_3380insT (GRCh37/hg19 chr4:184633774), that is absent in both the gnomAD and ExAC control population, with a CADD PHRED score of 35.0, and results in a protein change of p.Asp1127Valfs*47 (Table 2). Both variants were confirmed by Sanger sequencing and were inherited each from one of the unaffected parents (Fig. 1c).

**Table 2:** *TRAPPC11* variants and frequency for the newly-reported alleles.

Subject	Variant	Allele frequency	CADD v1.3
S1	NM_021942.5: c.1287 + 5G > A: p.Ala372_Ser429del	15/282698 (gnomAD) 3/121132 (ExAC)	15.64
S1	NM_021942.5:c.3379_3380insT: p.Asp1127Valfs*47	0 (gnomAD) 0 (ExAC)	35.0
S2	NM_021942.5:c.2938G > A; p.Gly980Arg	21/282732 (gnomAD) 5/121286 (ExAC)	34.0
S3	NM_021942.5:c.2938G > A; p.Gly980Arg		

The PHRED-like scaled C-score ranks a genetic variant relative to all possible substitutions of the human genome. A scaled C-score of 20 or greater indicates that the variant is amongst the 1% most deleterious substitutions^24^.

**Figure 2:**
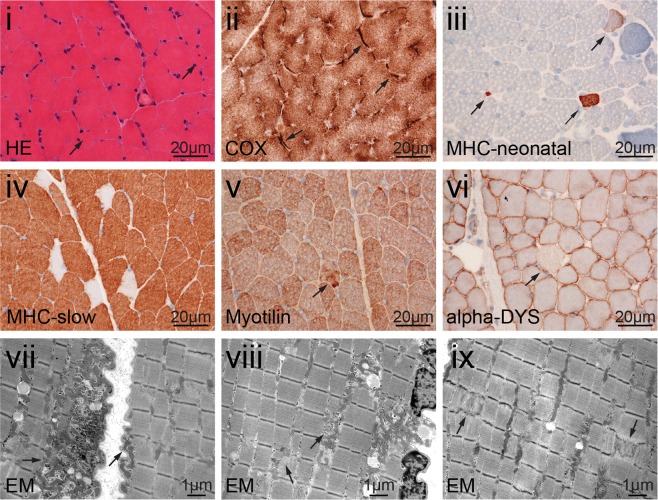
Muscle abnormalities in subject S2 at age 8 years. (i) H&E stained sections show muscle fibers with increased variation of diameter and few fibers with internalized nuclei (arrows). (ii) In the COX/SDH staining some fibers show subsarcolemmal mitochondrial aggregates (arrows). (iii) MHC-neonatal expression in few fibers indicates regenerative muscle fiber (arrows). (iv) Staining with MHC slow antibodies shows type1 fiber predominance. (v) Antibodies against myotilin display a few fibers with subtle myofibrillar disintegration (arrow). (vi) Sarcolemmal α-dystroglycan expression is reduced in a few fibers (arrow). Ultrastructural analysis shows that the sarcolemma is focally folded with subsarcolemmal accumulation of mitochondria (arrows) (vii) and intermyofibrillar accumulation of mitochondria (arrows) (viii), and focal Z-band abnormalities (arrows) (ix).

Subject S2 is the fourth of six children of healthy consanguineous Turkish parents. The girl was born at 26 weeks gestational age due to preterm premature rupture of membranes and amnion infection syndrome. Birth weight was 1000 g, length was 35 cm, and head circumference was 25 cm (10^th^–50^th^ centiles). The perinatal period was complicated by hyperbilirubinemia, pneumonia, and respiratory distress syndrome, and the subject had to be ventilated for 28 days. Muscular hypotonia and elevated CK values prompted a muscle biopsy at age 9 months that showed myopathic alterations. Her early motor development was delayed, and she achieved walking without support at age 3 years. At age 8 years, the subject was hospitalized due to pneumonia. At that age, weight was 15 kg (centile < 1, −3.2 SD), length was 122 cm (10^th^ centile), and head circumference was 46 cm (centile < 1, −3.1 SD). She presented with marked muscle atrophy, had difficulties when standing up from the ground (Gowers sign), and showed a waddling gait, hyperlordosis, and scoliosis. A second muscle biopsy showed features of a congenital myopathy with type 1 fiber predominance and focal Z-band abnormalities at the ultrastructural level (Fig. 2). Neuropsychological testing disclosed a learning disability (IQ 75). She had normal aspartate aminotransferase (GOT) values, but elevated alanine aminotransferase (GPT) values (maximum 118 U/l, normal range < 35). Her CK was elevated with values up to 500–1500 U/l. While albumin and coagulation parameters were within the normal range, cholinesterase values were reduced (minimum 606 U/l, normal range of 4000–12000). Moreover, amylase and lipase values were slightly elevated on several occasions without any clinical signs of pancreatic dysfunction. Echocardiography showed no signs of cardiac involvement.

The subject lost ambulation at age 10 years. Progression of scoliosis necessitated spinal fusion at age 11 years, and recurrent pneumonias and declining lung function resulted in tracheostomy and invasive ventilation at age 14 years. Since then, the patient’s clinical status has been stable. At last follow-up, at age 23 years, she was ventilated 20 hours per day, fed by a gastric tube, but was able to raise her arms against gravity. A diagnosis of TRAPPC11-related limb girdle muscular dystrophy 2S (LGMD2S) was made by whole exome sequencing (MYO-SEQ project) that revealed a homozygous variant c.2938G > A; p.Gly980Arg. This variant is rare in both gnomAD and ExAC (0.007% and 0.004%, respectively, with no homozygotes) with a CADD PHRED score of 34.0 (Table 2).

Subject S3 is the eldest of three children of consanguineous Syrian parents. She was born at term without complications. Birth weight was 3200 g, length was 50 cm, and head circumference was 35 cm (all values 25^th^–50^th^ centile). Her early motor development was delayed, and she achieved free walking at age 2.5 years. She developed a proximal muscle weakness with difficulties when rising from the floor after age 5 years. At age 10 years, her walking distance was reduced to about 100 m, and since age 25 years, she is only able to walk a few steps. At last follow-up, at age 32 years, her condition was stable.

**Figure 3:**
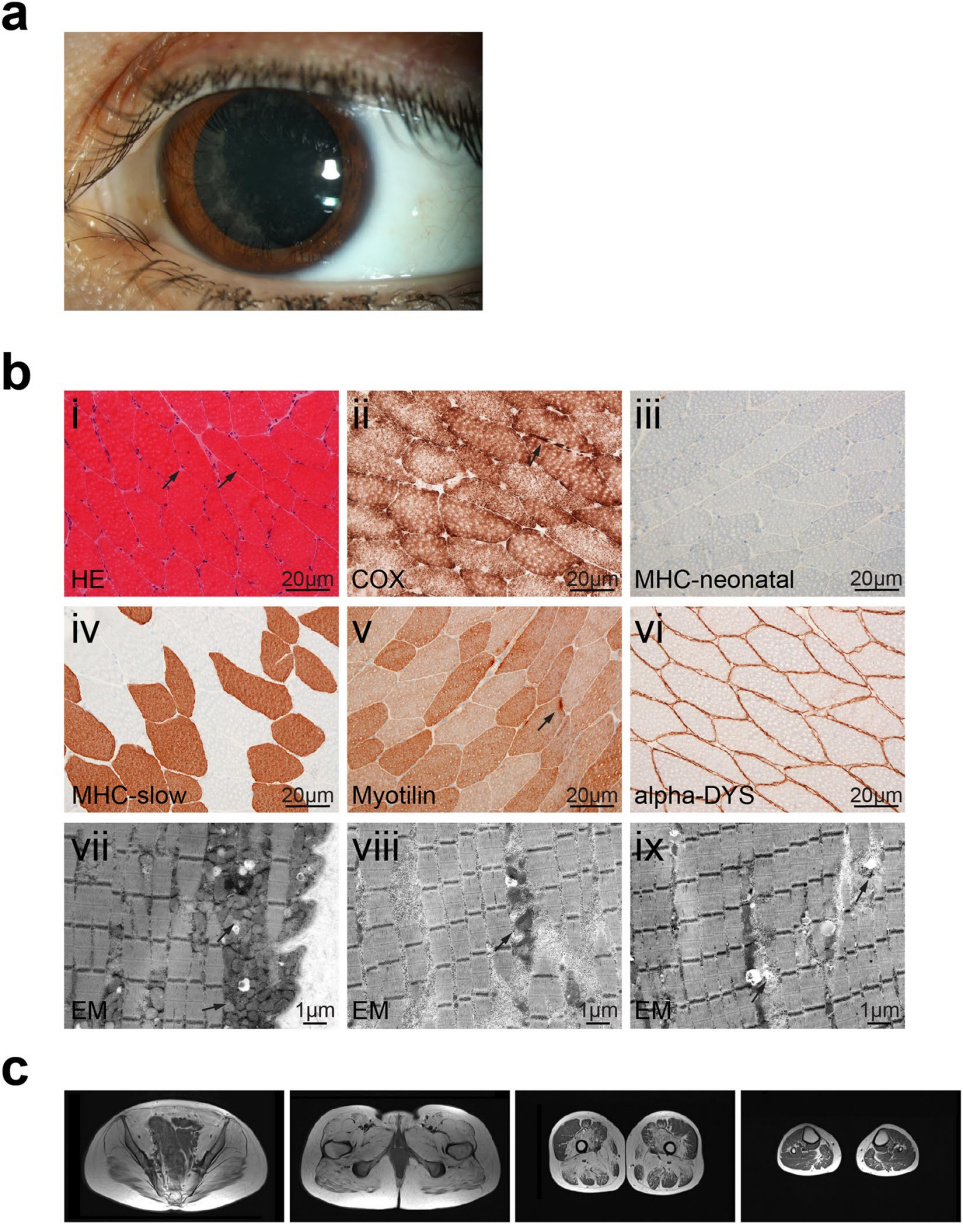
Eye and muscle abnormalities in subject S3 at age 27 years. (**a**) Eye examination displays non-progressive cataracta coerulea. (**b**) (i) H&E stained sections show muscle fibers with minor variation in diameter and few internal nuclei (arrows). (ii) COX/SDH staining depicts some fibers with little subsarcolemnal mitochondrial aggregates (arrow). (iii) No upregulation is seen when using antibodies against MHC neonatal in the muscle fibers. (iv) Staining with antibodies against MHC slow shows normal distribution of type 1 or 2 fibers. (v) A few muscle fibers show myofibrillar disintegration with antibodies against myotilin (arrow). (vi) Sarcolemmal α-dystroglycan expression is normal. (vii) Ultrastructural analysis shows that the sarcolemma is focally folded with moderate subsarcolemmal accumulation of mitochondria (arrows). The myofibrillar architecture shows focal mild alterations with mitochondrial aggregates and extralysosomal glycogen (arrow) (viii) and focal unspecific loss of z-bands (arrows) (ix). (**c**) T1-weighted muscle MR images of S3 at age 30 years showing marked replacement of all pelvic muscles (gluteus maximus, iliacus, obturator internus, pectineus, and psoas muscles) by fat and connective tissue. At the upper thigh level, the vastus intermedius, the abductor magnus and brevis muscles were also severely affected whereas the vastus lateralis muscle was relatively spared. Similarly, the lower thigh muscles appeared well preserved.

She had normal GOT values, but moderately increased GPT values (maximum 186 U/l). Her CK was elevated up to 600–1800 U/l. In addition, amylase and lipase values were slightly elevated on several occasions without any clinical signs of pancreatic dysfunction. Her cholinesterase values were normal. Echocardiography performed several times during the course of disease was always normal, whereas her vital capacity (VC) was reduced to approximately 50%, but she has not required ventilation. An ophthalmological examination revealed a bilateral cataract (Fig. 3a). A psychological examination at age 18 years yielded an IQ of 85, and cranial MRI at age 32 years showed a mild cortical atrophy. A muscle biopsy taken at age 27 years showed muscle fibers with features of an unspecific myopathy (Fig. 3b) and an MRI displayed fatty muscle degeneration (Fig. 3c). Diagnosis of TRAPPC11-related LGMD2S was established by whole exome sequencing that revealed the homozygous variant c.2938G > A; p.Gly980Arg^15^.

### Fibroblasts from subjects S1, S2 and S3 have membrane trafficking defects

**Figure 4:**
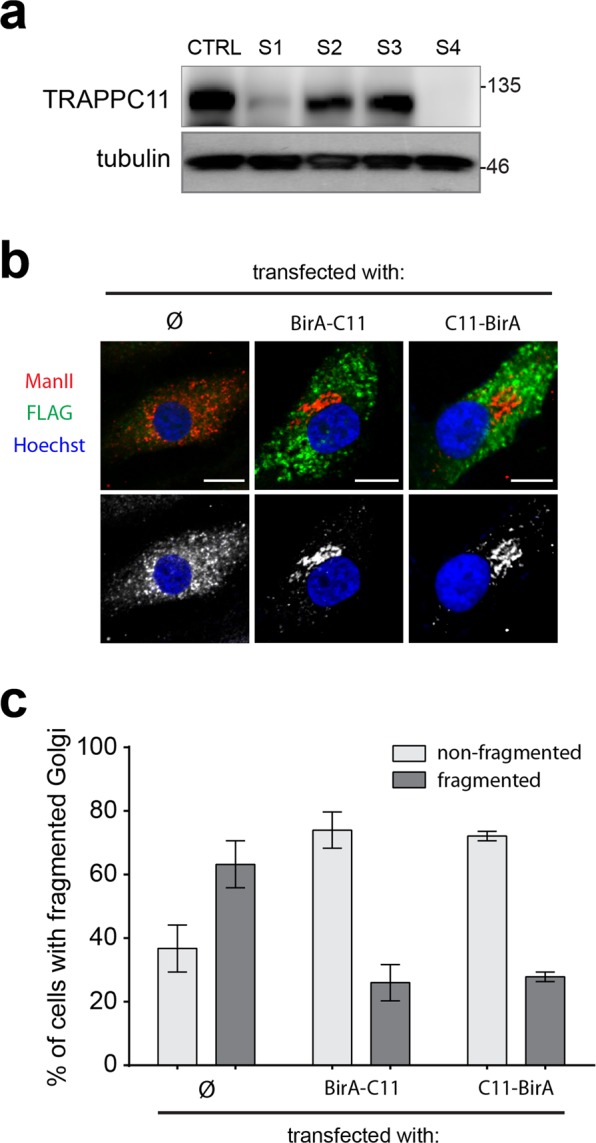
Golgi morphology is altered in fibroblasts from individual S1. (**a**) Lysates were prepared from control, S1, S2 and S3 fibroblasts and probed for TRAPPC11 and tubulin (as a loading control). Molecular size standards are indicated to the right of the blots. Note that the tubulin and TRAPPC11 blots are from the same gel. The full, uncropped western blot is shown in Fig. S1. (**b**) Fibroblasts from S1 were untransfected (ø), fixed and stained with antibodies recognizing the Golgi marker mannosidase II (red) or transfected with a TRAPPC11 construct fused at the amino terminus or the carboxy terminus to the protein BirA (green) (BirA-C11 and C11-BirA, respectively). The BirA fusions contained a FLAG epitope tag to allow for identification of transfected cells. Note the altered morphology of the Golgi in fibroblasts from the affected individual that is rescued by either TRAPPC11 construct. The non-merged image is shown in the lower panel with the Golgi in white. (**c**) Quantification of the images from panel (**b**) was performed using criteria previously established^27,43^. N values ranged from 25 to 38 in a single rescue experiment. Error bars indicate SEM.

TRAPPC11 was first identified as a protein that associates with the TRAPP III membrane trafficking complex^1^. Depletion of the protein in HeLa cells resulted in Golgi fragmentation. Previous studies have suggested that some variants result in reduced levels of the protein^15,17,19^, possibly leading to morphological changes in the Golgi. We found reduced levels of TRAPPC11 in S1 (Fig. 4a), consistent with the unstable protein generated by the c.1287 + 5G > A allele as previously reported (subject S4, Fig. 4a)^15^. In contrast, S2 and S3 had levels of the protein comparable to that of control (Fig. 4a). Consistent with reduced TRAPPC11 levels, fibroblasts from S1 displayed severe Golgi morphological alterations resulting in highly fragmented Golgi membranes (Fig. 4b,c). The Golgi alterations could be rescued by transfection of the cells with wild-type TRAPPC11 fused at either the amino- or carboxy-terminus to the biotin ligase BirA (Fig. 4b,c), confirming that the Golgi morphological alterations were TRAPPC11-dependent. The BirA served as a steric impediment for the function of these ends of the protein as recently shown^6^. Thus, neither the carboxy- nor amino-terminus of TRAPPC11 is critical for its function in maintaining normal Golgi morphology.

**Figure 5:**
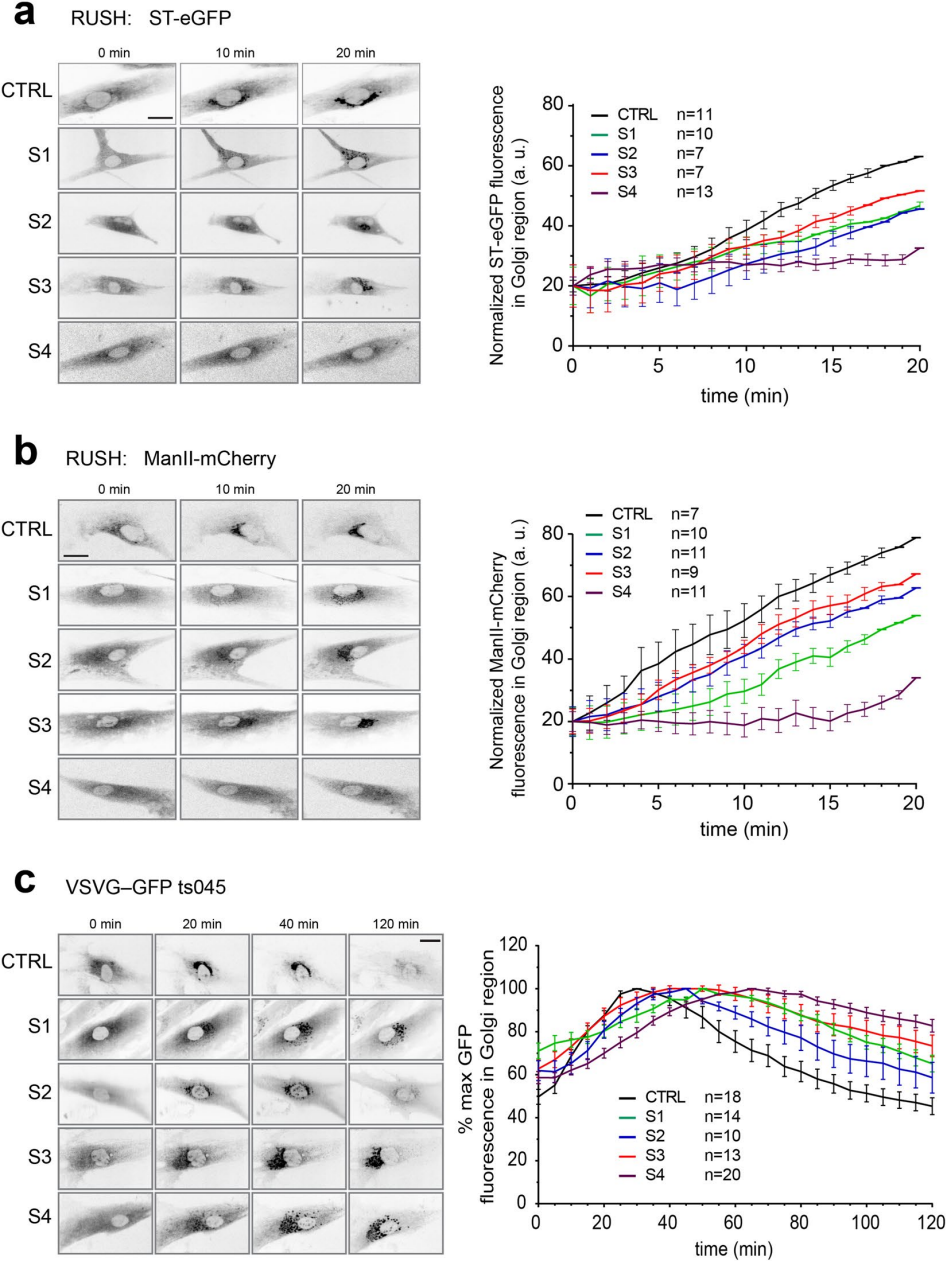
Trafficking from the ER to the Golgi and exit from the Golgi is delayed in fibroblasts from individuals S1, S2 and S3. The RUSH assay was performed on fibroblasts from control, S1, S2, S3 and S4 using (**a**) ST-eGFP and (**b**) mannosidase II (ManII)-mCherry. The cells were imaged every minute over a period of 20 minutes after the addition of biotin to induce release of the cargo molecule from the endoplasmic reticulum. Fluorescence in the Golgi region was quantified as described in Koehler *et al*.^17^ and is displayed to the right of the representative images. (**c**) Fibroblasts from control, subjects S1, S2, S3 and S4 were infected with virus expressing VSVG-GFP ts045. After 1 hour, the cells were shifted to the restrictive temperature of 40 °C overnight. Prior to imaging the cells, cycloheximide was added. The cells were then imaged every minute over a 2-hour period at 32 °C. Fluorescence in the Golgi region was quantified for the N values indicated over at least two independent experiments as described above and is displayed to the right of the representative images. The scale bars represent 25 µm and the error bars represent SEM.

We then examined the fibroblasts for membrane trafficking defects using the RUSH assay^25^. In this assay, a fluorescent cargo protein is retained in the ER until the cells are exposed to biotin. The cargo (ST-eGFP and ManII-mCherry) is then monitored as it exits the ER and arrives in the Golgi. As shown in Fig. 5a,b, fibroblasts from all three subjects of this study as well as subject S4 displayed a delay in arrival of both cargo proteins at the Golgi compared to control. We then examined the trafficking of a marker protein (VSVG-GFP ts045) that traverses the biosynthetic pathway, allowing for assessment of cargo arrival and release from the Golgi^26^. In this assay, VSVG-GFP ts045 is retained in the ER at restrictive temperature (40 °C) and released upon downshift to permissive temperature (32 °C). In fibroblasts from all three subjects as well as S4, we noted a delay in arrival of the protein at the Golgi, consistent with the results of the RUSH assay, as well as a defect in release of the protein from the Golgi (Fig. 5c). Based on these two assays we conclude that fibroblasts from all three subjects display membrane trafficking defects along the biosynthetic pathway.

### Fibroblasts from subjects S1, S2 and S3 display a defect in protein glycosylation

**Figure 6:**
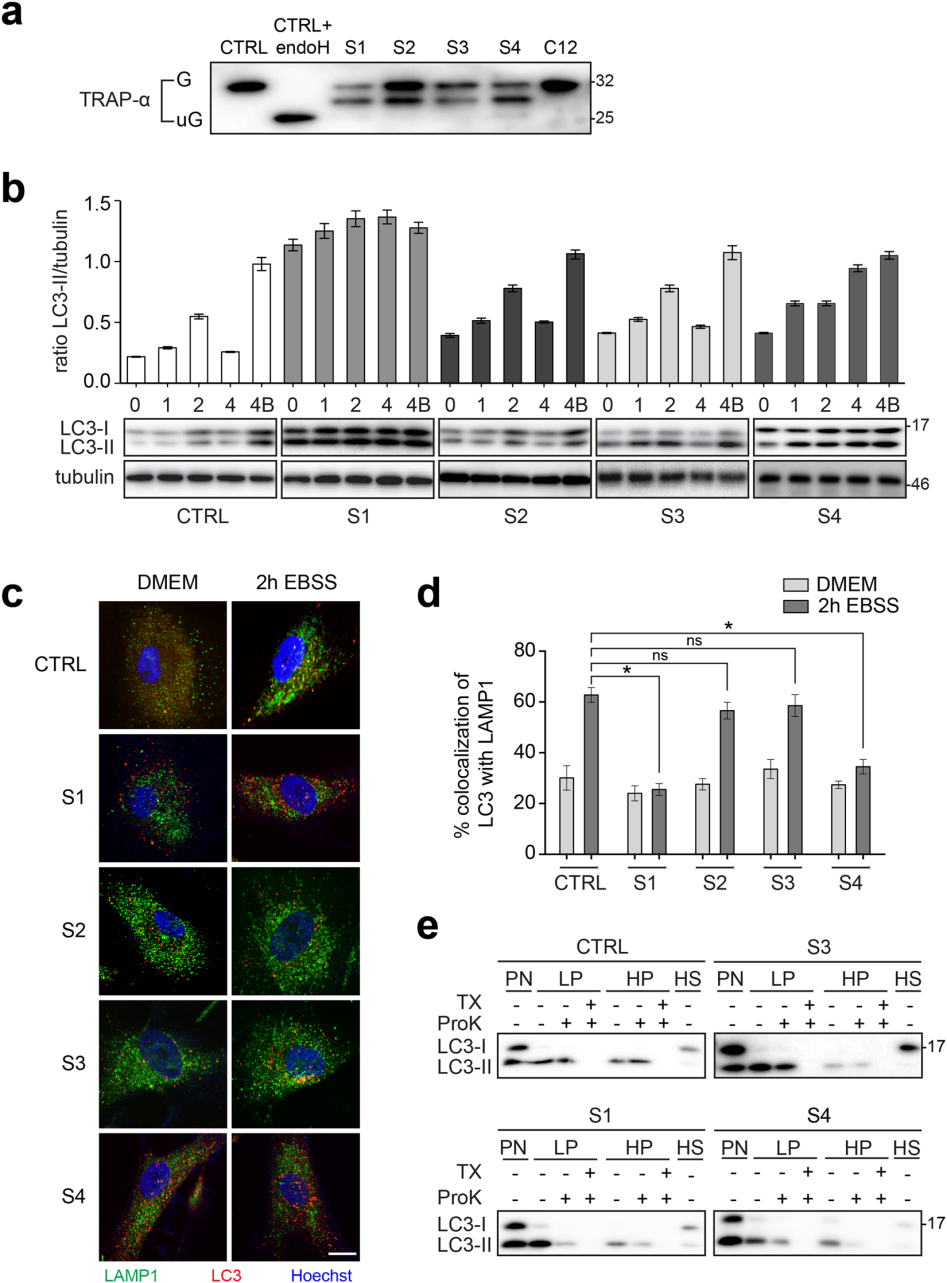
Assessment of glycosylation and autophagic flux defects in fibroblasts from individuals S1, S2, S3 and S4. (**a**) Lysates were prepared from fibroblasts from control, subjects S1, S2, S3 and S4 (an individual with a homozygous c.1287 + 5G > A variant^15^) as well as an individual with a homozygous c.145delG variant in TRAPPC12^27^ (C12). The lysates were fractionated by SDS-PAGE, transferred to PVDF membrane and probed for the ER-resident glycoprotein TRAP-α. A portion of the lysate from control cells was treated with endoglycosidase H to convert the glycosylated (G) form of the protein to the unglycosylated form (uG). (**b**) Fibroblasts from control, subjects S1, S2, S3 and S4 were left in nutrient rich medium (0) or starved for 1, 2 or 4 hours. One sample was starved for 4 hours in the presence of bafilomycin A1 (4B). Lysates were prepared and probed for LC3 and tubulin as a loading control. The graph shows the ratio of the autophagy-specific (LC3-II) form of the protein normalized to tubulin. In all cases, the tubulin blots are from the same gel as the LC3B blots. Note that samples from control and S2 were fractionated on the same gel, while S1, S3 and S4 were from different gels. Error bars indicate SEM. A representative blot from a minimum of three replicates is shown below the graph. (**c**) Fibroblasts from control, subjects S1, S2, S3 and S4 were left either in nutrient-rich medium or starved for 2 hours. The cells were then fixed and immunostained for LC3 and LAMP1. (**d**) Co-localization between LC3 and LAMP1 from the cells in panel (**c**) was quantified using Imaris. Statistical significance was assessed using a one-way ANOVA with posthoc Tukey HSD analysis. The asterisk (*) indicates p < 0.0001. (**e**) Fibroblasts from control, subjects S1, S3 and S4 were starved for 2 hours in the presence of bafilomycin A1. Lysates were prepared (PN) and fractionated into low speed (LP), high speed (HP) pellets and a high speed supernatant (HS) fraction as described in the methods section. The fractions were subjected to proteinase K (ProK) with or without pre-treatment with the detergent Triton X-100 (TX) and then probed for LC3. Each sample was fractionated on a separate gel. Molecular size standards are indicated to the right of the blots in panels (**a,b,e**). The full, uncropped western blots for these panels are shown in Fig. S1.

A previous study using mainly zebrafish as a model system suggested that TRAPPC11, to the exclusion of other TRAPP-associated proteins, plays an undetermined role in protein *N*-glycosylation^11^. When examined in HeLa cells, depletion of TRAPPC11 resulted in defective glycosylation of TRAP- α, a resident ER protein whose glycosylation is independent of membrane trafficking. We therefore examined the fibroblasts from S1, S2, S3 and S4 for the glycosylation state of TRAP-α. The inclusion of S4 in this and subsequent analyses allows us to assess whether the c.3379–3380InsT allele in subject S1 is sufficient for normal TRAPPC11 function. As shown in Fig. 6a, fibroblasts from all four subjects showed a form of TRAP-α that migrated between the fully and the unglycosylated forms of the protein in addition to a form that co-migrated with the fully glycosylated protein. The partially glycosylated form was not seen in fibroblasts from an individual with bi-allelic variants in TRAPPC12^27^, supporting our previous work in HeLa cells that indicates this is specific to defects in TRAPPC11^11^. These results demonstrate that, similar to a partial depletion of TRAPPC11 in HeLa cells, the TRAPPC11 variants reported herein also affect protein glycosylation.

Glycosylation defects can result in a buildup of proteins in the ER leading to ER stress, which can manifest by upregulation of a set of genes including *ATF6*, *XBP1*, *DDIT3*, *BIP*, *DNAJC3*, *EDEM1* and *ATF4* responsible for a particular class of the unfolded protein response^28^. ER stress is also associated with proteolytic processing of the ATF6 transcription factor to a faster-migrating species^29^. We therefore examined fibroblasts from S1, S2, S3 and S4 for signs of ER stress by both qPCR and ATF6 processing. Except for *XBP1* expression which showed a modest increase in subject S1 of 2.70 ± 0.49 fold compared to control, no other genes examined showed any increase (not shown). Furthermore, ATF6 processing did not reveal any significant differences when compared to control (not shown). Therefore, unlike the zebrafish *trappc11* mutant model system^11^, we conclude that the bi-allelic *TRAPPC11* variants reported herein result in a glycosylation defect in fibroblasts but do not result in an increase in expression levels of genes that respond to ER stress.

### Fibroblasts from subjects S1 and S4 display a defect in autophagic flux

We have recently shown that TRAPPC11 functions in an early stage of autophagy, after the formation of nascent isolation membranes^6^. Therefore, we examined the fibroblasts from all three subjects as well as S4 for defects in autophagic flux as measured by the starvation-induced accumulation and then disappearance of the autophagic marker LC3-II. As shown in Fig. 6b, the fibroblasts from S1 and S4, but not S2 nor S3, displayed a defect in autophagic flux since LC3-II could not be cleared from the cells over the time course of starvation examined. The fibroblasts from S1 also displayed a high level of LC3-II even prior to starvation-induced autophagy. Although the accumulation of LC3-II prior to starvation was not as dramatic in S2, S3 and S4 as compared to S1, the levels were nevertheless elevated in the fibroblasts derived from these three subjects.

In order to confirm the autophagic flux defect, we examined the fibroblasts for co-localization between the autophagosome marker LC3 and the lysosome marker LAMP1. Co-localization would suggest that the formation of autolysosomes, organelles resulting from the fusion of autophagosomes with lysosomes, has taken place. Control fibroblasts showed a relatively high level of starvation-dependent co-localization between the marker proteins, as did fibroblasts from S2 and S3 (Fig. 6c,d). Consistent with the autophagic flux defect in S1, fibroblasts from this individual showed poor starvation-dependent co-localization. The same was noted for subject S4.

Using a protease protection assay^30^, we recently demonstrated that the starvation-dependent accumulation of LC3 punctae in S1 were in fact unsealed isolation membranes^6^. This assay separates lysates (PN) into two different membrane fractions (LP and HP) and a cytosolic fraction (HS). The fractions are treated with protease K either with or without detergent. If the autophagosomal marker LC3-II is susceptible to digestion in the absence of detergent, then the autophagosome membranes are not considered to be sealed. Employing this assay, we showed that, consistent with the autophagic flux and co-localization data above, fibroblasts from S3 are not defective in producing sealed autophagosomes since membrane-associated LC3 was only susceptible to protease digestion in the presence of detergent, similar to what was seen in control (Fig. 6e). In contrast, and similar to fibroblasts derived from S1, fibroblasts from S4 in which full-length TRAPPC11 is absent also failed to seal isolation membranes into autophagosomes since LC3-II was susceptible to proteinase K treatment in the absence of detergent. Collectively, our data suggest that the extreme carboxy-terminus of TRAPPC11, but not residue Gly980, is critical for starvation-induced autophagy.

## Discussion

It is becoming increasingly clear that proteins described as TRAPP subunits and thought to be stable components of a mammalian TRAPP complex, may not in fact be stably associated with the complex. This is in contrast to the yeast TRAPP proteins that seem to be stably associated with either TRAPP II or TRAPP III^31–34^, and only break down into the likely artifactual TRAPP I upon incubation in high salt^35^. The implication of this idea is that these TRAPP-associated proteins are capable of functioning independently of the complex. This is supported by the non-identical, but partially overlapping clinical phenotypes associated with TRAPP protein variants (reviewed in^14^). Further support comes from the fact that several proteins including TRAPPC2, TRAPPC9, TRAPPC11 and TRAPPC12 have been uniquely implicated in collagen export, NF-κB signaling, *N*-linked protein glycosylation and chromosome congression, respectively, in addition to a role in membrane trafficking^11,12,36,37^. Even within TRAPP III, the complex-specific proteins TRAPPC8, TRAPPC11 and TRAPPC12 appear to have differing functions^6^. Thus, in order to better understand how these proteins can function in multiple and shared cellular processes it is necessary to identify regions of the proteins that are critical for their participation in a specific process.

**Figure 7:**
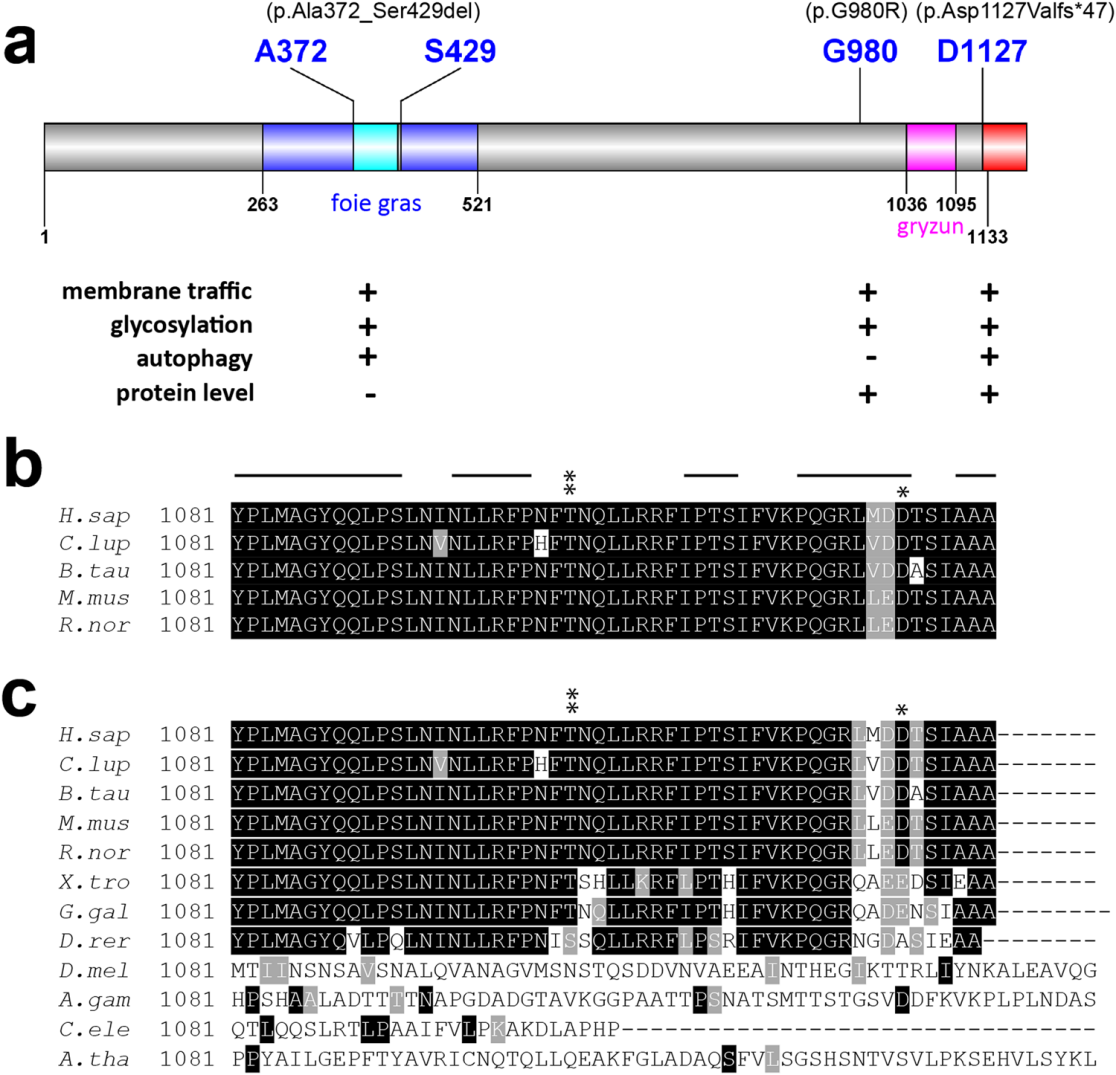
Functions associated with variants in subjects S1, S2 and S3, and the conservation of the carboxy terminus of the protein. (**a**) Domain topology of TRAPPC11 showing the location and the variants described in this study. The foie gras domain (amino acids 263–521) is shaded in blue and the deletion resulting from the c.1287 + 5G > A variant is shaded in cyan. The first altered residue resulting from the c.3379_3380insT variant is indicated in blue and the resulting extension of the carboxy terminus is indicated in red. The gryzun domain is shaded in pink. A summary of the assay results and protein levels is indicated below the cartoon. For the assays, “+” indicates the assay was affected by the variant and “−” indicates it was not. For the protein levels, “+” indicates the presence of the protein and “−” indicates its absence. (**b**,**c**) A multiple sequence alignment of the carboxy terminus of TRAPPC11 (starting from amino acid 1081) using (**b**) *H*.*sapiens*, *R*.*norvegicus*, *M*.*musculus*, *B*.*taurus* and *C*.*lupus* or (**c**) the former species as well as *X*.*tropicalis*, *G*.*galus*, *D*.*rerio*, *D*.*melanogaster*, *A*.*gambiae*, C.*elegans* and *A*.*thaliana*. Black shading represents identities in at least 4 species in (**b**) and 9 species in (**c**) and gray represents similarities in at least 6 species in (**c**). The asterisks indicate the position of two TRAPPC11 variants that have been described in this region from the present report and in Matalonga *et al*.^20^. The lines above the sequences in panel (**b**) indicate predicted α-helices in the human sequence using JPred4.

We have described an individual with novel compound heterozygous *TRAPPC11* variants as well as two individuals with identical homozygous p.Gly980Arg variants that allow us to speculate on important regions of the protein that mediate different cellular processes (Fig. 7a). The severity of the muscle weakness and the course of the disease differed distinctly between the two subjects harboring the same homozygous mutation. Therefore, it appeared meaningful to analyse the pathophysiological effects of the genetic defect in both of them. Using fibroblasts derived from these individuals, we have implicated Gly980 as part of a region critical for both membrane trafficking and *N*-linked glycosylation, but not in the maintenance of normal Golgi morphology. The Gly980 residue also does not appear to be crucial for autophagy. A multisequence alignment revealed that this region of the protein is highly conserved and that this residue in particular is invariant^15^. Expansion of the alignment to include more phylogenetically distant organisms confirms that this residue is indeed invariant (not shown). Although this variant was reported to likely affect protein structure, this may be very localized as it does not affect autophagy, a process that is believed to be linked to the extreme carboxy terminus (see below).

Subject S1 differs clinically from S2 and S3 mainly because of the presence of spasticity, hyperkinesia and choreiform movements. However, these clinical signs have been previously reported separately or in combination in other individuals with TRAPPC11 variants (including the c.1287 + 5G > A variant) and in individuals with variants in other TRAPP proteins (TRAPPC9 and TRAPPC12) as recently reviewed^14^. Thus, some of these clinical features may be related to the function of TRAPPC11 proteins in their corresponding TRAPP complexes and to the expression of these proteins in the central nervous system. Furthermore, the impairment of autophagy observed only in S1 and associated with the c.3379_3380insT mutation may also contribute to the spasticity and choreiform movements since these clinical signs are characteristic of other human diseases in which autophagy plays a significant role (e.g. hereditary spastic paraparesis due to mutations in TECPR2 and SPASTIZIN^38^, and Huntington’s disease^39^).

With respect to the compound heterozygous case, the variant c.1287 + 5G > A (p.Ala372_Ser429del) was previously described in homozygous form^15,40^. The resulting protein appeared to be unstable as it was not detected by western analysis. One study demonstrated that this variant affects membrane trafficking^15^. Here we also demonstrated that this variant affects formation of autophagosomes and results in an *N*-linked glycosylation defect. These results are not unexpected considering that the protein levels of this variant are undetectable. The compound heterozygous individual also displays defects in all three processes (membrane trafficking, autophagy and *N*-linked glycosylation) as well as in maintenance of normal Golgi morphology, implicating the extreme carboxy terminus of the protein in its functions. This is based on the fact that full-length TRAPPC11 is still detectable in this individual, likely reflecting the p.Asp1127Valfs*47 protein. Further support for the importance of the extreme carboxy terminus comes from the absence of this allele in both gnomAD and ExAC. A multisequence alignment limited to humans, rodents, canine and bovine reveals that the carboxy terminus, like the rest of TRAPPC11, is well conserved (Fig. 7b). When more phylogenetically distant organisms are included, the conservation of this region of the protein begins to drop (Fig. 7c). Thus, we speculate that higher eukaryotes may have evolved a unique autophagy-related function in the carboxy terminus of the protein. It remains to be seen if mutations at the extreme carboxy terminus of the TRAPPC11 protein in these phylogenetically distant organisms will affect autophagy. Recent work from our laboratory suggests that this region of the protein is important for recruiting the ATG2B-WIPI4 complex^6^, a factor that is required for the sealing of isolation membranes into enclosed autophagosomes^30^.

TRAPPC11 dysfunction has been implicated in protein *N*-glycosylation. The first report came from a study using a zebrafish *trappc11* mutant which displayed reduced levels of lipid-linked oligosaccharides and upregulation of genes necessary for terpenoid biosynthesis, a precursor for the carbohydrate carrier dolichol^11^. This same study was also the first to implicate human TRAPPC11 in protein *N*-glycosylation. Two recent reports have suggested that naturally-occurring *TRAPPC11* variants are associated with defective glycosylation of the muscle protein alpha-dystroglycan^18^ and altered glycosylation of both serum transferrin and apoCIII^20^. Here, we have extended the glycosylation defect to three more *TRAPPC11* variants (subjects S1, S2/S3 and S4), suggesting that this may be a hallmark of TRAPPC11 dysfunction. While one variant in this study (subject S4) has reduced levels of the protein^15^, subject S1 does not show the same. Instead, this subject has a variant near the carboxy terminus of TRAPPC11. The affected region of the protein is similar to a previously published TRAPPC11 variant (p.Thr1104Ala) that was reported to have glycosylation defects^20^, though TRAPPC11 protein levels were not reported. It is noteworthy that the latter variant also displayed a modest (2–2.5 fold) increase in the expression levels of four genes involved in the unfolded protein response (UPR), including *XBP1* which was also modestly elevated in our study. Thus, activation of UPR may be correlated to the extent of the glycosylation defect, an idea that requires further study. In the three subjects reported herein, the glycosylation defect seen in the fibroblasts might be mild since a substantial amount of fully glycosylated protein is also detected in all three subjects.

We considered the possibility that the carboxy-terminal extension of 46 residues might create a motif that could alter the function of the resulting protein. To this end we first scanned these residues in the Eukaryotic Linear Motif (ELM) database. As expected, we identified a number of potential phosphorylation sites, kinase- and phosphatase-interacting motifs, and an *N*-linked glycosylation motif. Such motifs are not unexpected and none of them could help explain the membrane trafficking or autophagic flux defects that we reported. It should be stressed that we recently showed that only an amino-terminal tag on TRAPPC11, and not a carboxy-terminal tag, could rescue a TRAPPC11-dependent autophagy defect^6^, further highlighting the importance of the unblocked C-terminal end of TRAPPC11.

With the identification of several new individuals harboring variants in TRAPPC11, the present study strengthens the relationship between dysfunction of this gene and pathophysiology, and implicates various regions as critical for the normal function of the protein. There are now five reported variants within the foie gras domain^9,15,18–20,40^, suggesting that this domain is important for the function of the protein. Identification of subject S2 makes a total of three published individuals with a G980 variant^15,19^, and here we show that this residue is important for membrane trafficking and protein glycosylation but not for autophagy. Finally, the variants in subject S1 suggest an important role for the extreme carboxy terminus of the protein. Considering that there are two other reports of individuals having variants close to the carboxy terminus at residues Thr1104^20^ or Pro1005^9^, the importance of this region of the 1133 amino acid-long protein may be extended to the final ~130 amino acids, overlapping with the gryzun domain. While a function for the carboxy terminus of the protein in autophagy has recently been elucidated^6^, how this region affects membrane trafficking and protein glycosylation remains to be determined in future studies.

## Methods

This study was carried out in accordance with the Declaration of Helsinki. Written informed consent was obtained from patients and/or their parents or guardians. All experimental protocols were approved by the ethical committee of the Medical Faculty of the University of Giessen, Germany, and by the Comitè d'Ètica d’Investigació Clínica (CEIC) de la Fundació Sant Joan de Déu (Fundacion Sant Joan de Déu Ethics Committee on Clinical Research (CEIC)). Methods were carried out in accordance with the relevant guidelines and regulations.

### Molecular genetics

Whole exome sequencing, data processing and analysis were performed as previously described^41^ and confirmed by Sanger sequencing.

### Histology

Open muscle biopsies were taken from the lateral vastus muscles (S1, S2 and S3) or the deltoid muscle (S1), oriented and frozen according to standard procedures. Frozen sections (10 µm for S1 and 6 µm for S2 and S3 for histology and histochemistry, and 7 µm for immunohistochemistry) were examined using routine histological and histochemical stains including haematoxylin and eosin (HE) and modified Gomori trichrome and cytochrome oxidase (COX/SDH)^42^. Immunohistochemical analysis was performed using a Bench Mark XT automatic staining platform (Ventana, Heidelberg, Germany; Ultraview Universal DAB Detection kit) with the following primary antibodies: anti-MHC neonatal, anti-MHC slow, anti-Myotilin and anti-α-dystroglycan. The sections were examined on a Nikon Eclipse 80i microscope equipped with a CCD camera. For transmission electron microscopy (TEM) in S2 and S3, small biopsies were fixed in 6% glutaraldehyde/0.4 M phosphate buffered saline (PBS) and were processed with a Leica EM TP tissue processor. Ultrathin sections were contrasted with 3% lead citrate-3H_2_0 with a Leica EM AC20 (ultrastain kit II) and examined on a Zeiss EM109 TEM equipped with a sharp eye digital camera.

### Membrane trafficking assays

Fibroblasts were maintained in DMEM supplemented with 10% fetal bovine serum. In order to examine ER-to-Golgi trafficking, the RUSH assay was performed^25^ and quantified as previously described^27^. Trafficking of VSVG-GFP ts045 was performed following viral infection of the construct and quantified as previously described^17^.

### Autophagy assays

Assessment of autophagosome formation by susceptibility to proteinase K was performed essentially as described^30^ with minor modifications^6^. For assessment of autophagic flux the cells were washed with phosphate-buffered saline (PBS) and incubated with Earle’s Balanced Salt Solution (EBSS; Wisent, St. Bruno, Canada) for the times indicated in the figure. In some cases, 200 nM Bafilomycin A1 (Sigma-Aldrich) was included during a 2-hour starvation. The starved cells were returned to nutrient-rich medium by washing with PBS and incubating in DMEM with 10% FBS for 20 or 40 minutes. The cells were lysed by harvesting in lysis buffer (150 mM NaCl, 50 mM Tris pH 7.2, 1 mM DTT, 1% Triton X-100, 0.5 mM EDTA, Complete protease inhibitors (Roche, Basel, Switzerland)) and analyzed by western blotting for LC3 (abcam, Cambridge, MA). The data were normalized to the tubulin (Sigma-Aldrich, St. Louis, MO) signal developed from the same membrane as the LC3 blot.

### Antibodies

Primary antibodies used in this study were rabbit anti-LC3 (abcam, Cambridge, MA), mouse anti-LC3 (Santa Cruz, Dallas, TX), rabbit anti-LAMP1 (abcam, Cambridge, MA), rabbit anti-TRAPPC11 (Sacher laboratory), mouse anti-tubulin (Sigma-Aldrich, St. Louis, MO), rabbit anti-mannosidase II (gift from Dr. Kelly Moreman), mouse anti-TRAP-α (Santa Cruz, Dallas, TX), mouse anti-FLAG (Sigma-Aldrich, St. Louis, MO), anti-neonatal myosin heavy chain (NCL-MHCn;, Leica Biosystems, Wetzlar, Germany), anti-MHC neonatal (Novocastra/Leica Biosystems, Wetzlar, Germany) anti-MHC slow (Novocastra/Leica Biosystems, Wetzlar, Germany) anti-myotilin (Novocastra/Leica Biosystems, Wetzlar, Germany), and anti-alpha-dystroglycan (Merck-Millipore, Burlington, MA). Secondary antibodies used in this study were anti-mouse Alexafluor488 and anti-rabbit Alexafluor647 (Life Technologies, Carlsbad, CA). Note that for all western blots displayed, the membranes were cut in the region where the protein migrates in order to maximize the number of proteins that could be analyzed on each blot.

### Fluorescence microscopy

The cells were washed with PBS, fixed with 4% paraformaldehyde for 20 minutes at room temperature, quenched with 0.1 M glycine for 10 minutes and permeabilized with 0.1% Triton X-100 for 8 minutes. Blocking was performed in 5% normal goat serum in PBS for 30 minutes at room temperature. Primary antibodies were diluted in 5% normal goat serum and were added to coverslips and incubated overnight at 4 °C. Cells were washed two times 10 minutes with PBS. Secondary antibodies were diluted in 5% normal goat serum in PBS and applied for 1 hour at room temperature. The coverslips were washed two times for 10 minutes each with PBS with Hoescht being added during the first wash. The coverslips were then mounted with Prolong Gold AntiFade (Life Technologies, Carlsbad, CA) and sealed with nail polish. Images were recorded on an Olympus FV10i microscope fitted with a 60X objective, NA 1.35. The images were acquired with 0.25 µm increment size, deconvolved in AutoQuant (Media Cybernetics, Rockville, MD) and quantified with Imaris (Bitplane, Concord, MA). For colocalization of LC3 and LAMP1, a total of 10 cells were analyzed for each condition.

### Quantitative PCR

Messenger RNA (mRNA) was harvested from cells using the Quick-RNA Miniprep kit (Zymo Research, Irvine, CA) according to the manufacturer’s instructions. A total of 1 µg of mRNA was converted to cDNA using the First Strand cDNA Synthesis kit (OriGene Technologies, Inc., Rockville, MD). Polymerase chain reactions were then set up in triplicate using iTaq™ Universal SYBR® Green Supermix (Bio-Rad Laboratories, Hercules, CA). Samples were subjected to thermal cycling and detection of the fluorescent signal in a Bio-Rad CFX96 real-time PCR detection system. Quantification was performed by the ΔΔC_T_ method. Oligonucleotides used were: *ATF6*-F 5′ CAGACAGTACCAACGCTTATGCC-3′; *ATF6*-R 5′ GCAGAACTCCAGGTGCTTGAAG-3′; *XBP1*-F 5′-CTGCCAGAGATCGAAAGAAGGC-3′; *XBP1*-R 5′-CTCCTGGTTCTCAACTACAAGGC-3′; *DDIT3*-F 5′-GGTATGAGGACCTGCAAGAGGT-3′; DDIT3-R 5′-CTTGTGACCTCTGCTGGTTCTG-3′*; BIP*-F 5′-CTGTCCAGGCTGGTGTGCTCT-3′; *BIP*-R 5′-CTTGGTAGGCACCACTGTGTTC-3′; *DNAJC3*-F 5′-GGAGAGGATTTGCCACTGCTTTT-3′; *DNAJC3*-R 5′-CTCTGCTCGATCTTTCAGGGCA-3′; *EDEM1*-F 5′-ACGAGCAGTGAAAGCCCTTTGG-3′; *EDEM1*-R 5′-CCACTCTGCTTTCCAACCCAGT-3′; *ATF4*-F 5′-TTCTCCAGCGACAAGGCTAAGG-3′; *ATF4*-R 5′-CTCCAACATCCAATCTGTCCCG-3′; *ActB*-F 5′-CACCATTGGCAATGAGCGGTTC-3′; *ActB*-R 5′-AGGTCTTTGCGGATGTCCACGT-3′.

## Supplementary information

Figure S1

## Data Availability

All data generated or analysed during this study are included in this published article.
