# Publisher Correction: Characterization of three *TRAPPC11* variants suggests a critical role for the extreme carboxy terminus of the protein

**DOI:** 10.1038/s41598-020-76436-0

**Published:** 2020-11-10

**Authors:** Miroslav P. Milev, Daniela Stanga, Anne Schänzer, Andrés Nascimento, Djenann Saint-Dic, Carlos Ortez, Daniel Natera-de Benito, Desiré González Barrios, Jaume Colomer, Carmen Badosa, Cristina Jou, Pia Gallano, Lidia Gonzalez-Quereda, Ana Töpf, Katherine Johnson, Volker Straub, Andreas Hahn, Michael Sacher, Cecilia Jimenez-Mallebrera

**Affiliations:** 1 Department of Biology, Concordia University, Montreal, QC Canada; 2 Institute of Neuropathology, Justus Liebig University Giessen, Giessen, Germany; 3 Neuromuscular Unit, Neuropaediatrics Department, Hospital Sant Joan de Déu, Institut de Recerca Sant Joan de Déu, Barcelona, Spain; 4 U705 and U703 Center for Biomedical Research On Rare Diseases (CIBERER), Instituto de Salud Carlos III, Madrid, Spain; 5 Servicio de Pediatría, Hospital Universitario Nuestra Señora de Candelaria, Santa Cruz de Tenerife, Spain; 6 Pathology Department and Biobank, Hospital Sant Joan de Déu, Institut de Recerca Sant Joan de Déu, Barcelona, Spain; 7 Servicio de Genética, Hospital de La Santa Creu I Sant Pau, Barcelona, Spain; 8 The John Walton Muscular Dystrophy Research Centre, Institute of Genetic Medicine, Newcastle University and Newcastle Hospitals NHS Foundation Trust, Newcastle-upon-Tyne, UK; 9 Institute of Cellular Medicine, Newcastle University, Newcastle-upon-Tyne, UK; 10 Department of Child Neurology, Justus Liebig University Giessen, Giessen, Germany; 11 Department of Anatomy and Cell Biology, McGill University, Montreal, QC Canada

Correction to: *Scientific Reports* 10.1038/s41598-019-50415-6, published online 01 October 2019

In the original version of this Article, the author Daniel Natera-de Benito was incorrectly indexed. This error has now been corrected.

