# Supplementary material for: Characterization of three TRAPPC11 variants suggests a critical role for the extreme carboxy terminus of the protein
Source: Sci Rep. 2019 Oct 1;9:14036. doi: 10.1038/s41598-019-50415-6 (PMC6773699; doi:10.1038/s41598-019-50415-6)

Characterization of three *TRAPPC11* variants suggests a critical role for the extreme carboxy terminus of the protein

Miroslav P. Milev[1*], Daniela Stanga[1*], Anne Schänzer[2], Andrés Nascimento[3,4], Djenann Saint-Dic[1], Carlos Ortez[3], Daniel Natera-de Benito[3], Desiré González Barrios[5], Jaume Colomer[3], Carmen Badosa[3], Cristina Jou[4,6], Pia Gallano[4,7], Lidia Gonzalez-Quereda[4,7], Ana Töpf[8], Katherine Johnson[8,9], Volker Straub[8], Andreas Hahn[10†], Michael Sacher[1,11†], Cecilia Jimenez-Mallebrera[3,4†]

[1] Concordia University, Department of Biology, Montreal, Quebec, Canada

[2] Institute of Neuropathology, Justus Liebig University Giessen, Giessen, Germany

[3] Neuromuscular Unit, Neuropaediatrics Department, Hospital Sant Joan de Déu, Institut de Recerca Sant Joan de Déu. Barcelona, Spain

[4] U705 and U703 Center for Biomedical Research on Rare Diseases (CIBERER), Instituto de Salud Carlos III, Spain

[5] Servicio de Pediatría, Hospital Universitario Nuestra Señora de Candelaria, Santa Cruz de Tenerife, Spain

[6] Pathology Department and Biobank, Hospital Sant Joan de Déu, Institut de Recerca Sant Joan de Déu, Barcelona, Spain

[7] Servicio de Genética, Hospital de la Santa Creu i Sant Pau, Barcelona, Spain

[8] The John Walton Muscular Dystrophy Research Centre, Institute of Genetic Medicine, Newcastle University and Newcastle Hospitals NHS Foundation Trust, Newcastle-upon-Tyne, U.K.

[9] Institute of Cellular Medicine, Newcastle University, Newcastle-upon-Tyne, U.K.

[10] Department of Child Neurology, Justus Liebig University Giessen, Giessen, Germany

[11] McGill University, Department of Anatomy and Cell Biology, Montreal, Quebec, Canada

[†]address correspondence to cjimenezm@fsjd.org; michael.sacher@concordia.ca; Andreas.Hahn@paediat.med.uni-giessen.de

*equal contribution

Figure S1. Full blots for the western blots shown in figures 4 and 6. The regions cropped and used in the figures are indicated by red boxes. Molecular size standards are indicated next to each panel.

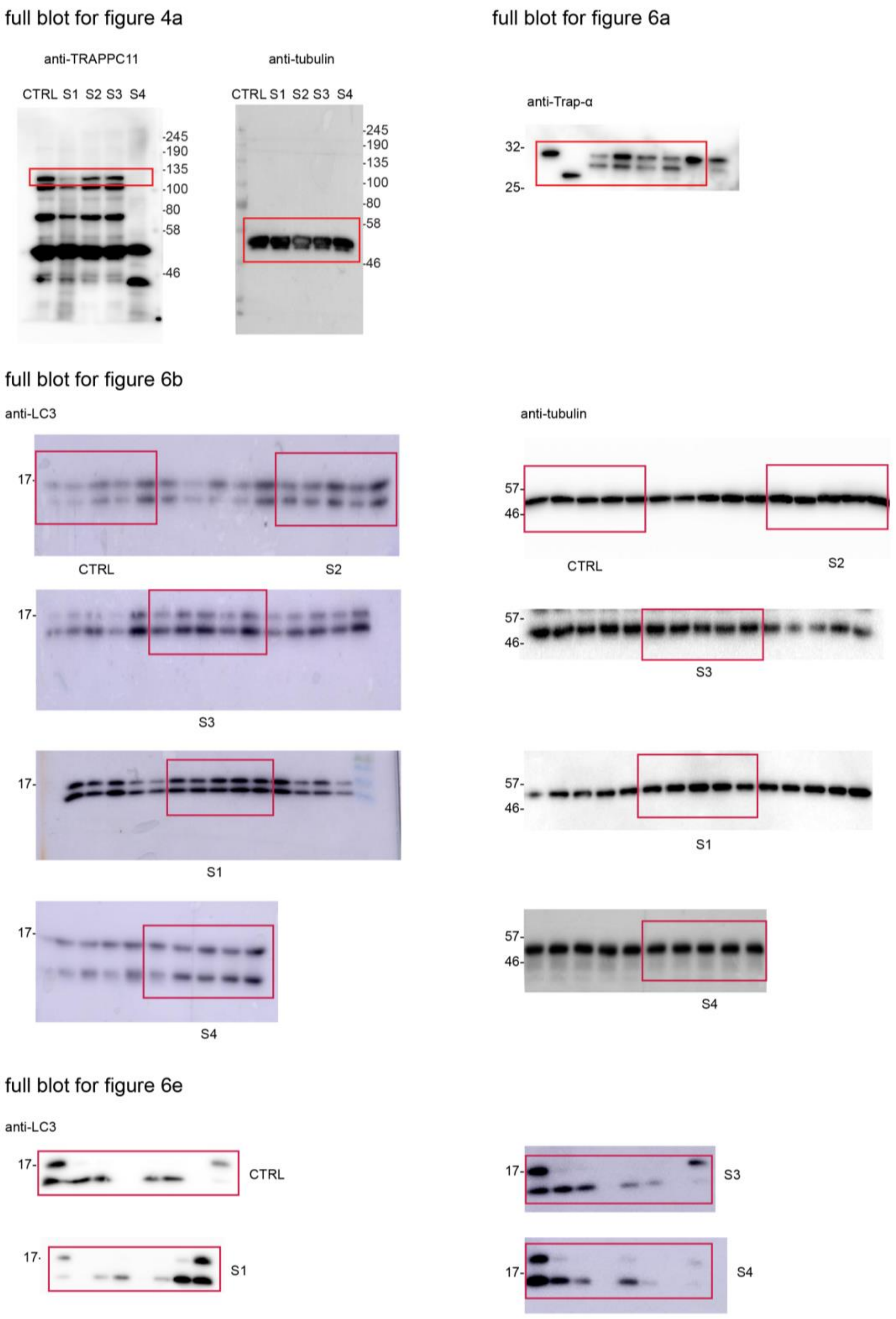

Supplement: Supplementary file 1 — Figure S1 [file 41598_2019_50415_MOESM1_ESM.pdf]
